# Immersive virtual reality in second-level education: a partnered narrative on the challenges and opportunities for STEM engagement

**DOI:** 10.1099/acmi.0.001028.v4

**Published:** 2025-07-22

**Authors:** Niall O'Leary, David Murphy, Ellen Condon, Danny Lonergan, Niamh Lordan, Deirdre Ní Théacháin, Caoimhín Ó Buachalla, Collette Uí Ghealbháin, Martin McHugh, Colm O'Hehir, F. Jerry Reen

**Affiliations:** 1 School of Microbiology, University College Cork, Cork, Ireland; 2 Environmental Research Institute, University College Cork, Cork, Ireland; 3 School of Computer Science and IT, University College Cork, Cork, Ireland; 4 Coláiste Nano Nagle, Limerick, Co. Limerick, Ireland; 5 Gaelscoil de hÍde, Mainistir Fhear Maí, Co. Chorcaí, Ireland; 6 Carrigtwohill Community College, Carrigtwohill, Co. Cork, Ireland; 7 Pobalscoil Chorca Dhuibhne, An Daingean Uí Chúis, Co. Chiarraí, Ireland; 8 Coláiste an Phiarsaigh, Gleann Maghair, Co. Chorcaí, Ireland; 9 Meánscoil San Nioclás, Rinn Ó gCuanach, Dún Garbhán, Co. Phort Láirge, Ireland; 10 SSPC, The Research Ireland Centre for Pharmaceuticals, Bernal Institute, University of Limerick, Limerick, Ireland; 11 SSPC, The Research Ireland Centre for Pharmaceuticals, University College Cork, Cork, Ireland

**Keywords:** biology, curriculum, immersion, second-level education, virtual reality

## Abstract

Digital education in the life sciences has seen several remarkable advances in recent years, not least with the advent of visual and immersive technologies that bring into focus the conceptually challenging abstract concepts that underpin molecular biology and the life sciences. In some cases, limitations in visualizing and modelling these concepts can prove to be a barrier to learning. Providing new entry points to learning through ‘doing’ or ‘seeing’ could prove to be a significant enhancer of engagement, unlocking hidden potential in our student cohorts and increasing the uptake of science as a subject of choice in higher education. In this study, second-level education teachers and higher education practitioners worked in partnership to explore the current state of the art around design and integration of immersive virtual reality simulations for the teaching of microbial and human cell structures in the classroom. We also considered the wider application of virtual reality and immersive learning technologies for science, technology, engineering and mathematics engagement and learning.

## Data Summary

No new data, tools, software or code have been generated or are required for our work to be reproduced.

## Introduction

The last decade has seen an ever-increasing focus on the use of digital technologies in the education sphere. Several systematic reviews have reported learning effectiveness and engagement as two key features of the immersive virtual reality (VR) experience [1,5]. Together, they point towards an inherent benefit of using immersive VR to deliver better results in achieving particular learning objectives. Evidence for this comes in several modalities, with some studies referring to low-level evidence in the form of Grading of Recommendations, Assessment, Development and Evaluation tools [1], while others operated through the lens of the interactive, constructive, active or passive mode framework on cognitive engagement [2,6]. Student engagement and enjoyment are often referred to in these reports, with the immersive and interactive nature of VR making it a useful tool for differentiated instruction catering for the diverse learning styles and needs of the students [7]. However, while considerable attention has been given to the development and use of immersive VR in higher education [1,8,11] and early childhood education [12], less focus has been placed on the classroom experience in second-level education (post-primary/high school). Post-primary education in Ireland consists of a Junior Cycle (JC, years 1–3) and Senior Cycle (SC, years 5–6), between which students have the option to take a Transition Year (TY) where they experience a wide range of educational instruction and work experience. Further studies are thus required to establish the challenges and opportunities associated with VR implementation in second-level classrooms to ensure that students entering higher education in a digital age have the appropriate tools through which to progress their science, technology, engineering and mathematics (STEM) journey and career in a digital future.

It is notable how few studies report on the practical integration of immersive VR within the curriculum across all education sectors [12]. To this end, Marougkas *et al.* performed important work in systematically reviewing 69 studies between 2012 and 2022 for insights into the use of immersive VR for educational objectives in the classroom [4]. Within this cross-sectional review, the majority of papers were published in 2019–2020, derived their data from undergraduate students (most frequently from chemistry and engineering backgrounds) and, for the most part, were sourced in the USA and Indonesia. Across all studies, the manipulation of artificial objects within a virtual space was found to be the most frequently utilized personalization technique, enabling users to view objects from a perspective that would not be possible in a real scenario. However, while positive learning impacts were highlighted in the review, the correlation between the student engagement and taxonomic learning goals has not been as clearly demonstrated, and this remains a key research focus of VR integration in all sectors of education. Furthermore, the lack of studies reporting on second-level (post-primary, post-K12) student experiences and discipline of (micro)biology highlights how significantly underexplored these are in the context of VR. Other reports have highlighted the use of design-related approaches in immersive VR, whereby the practical elements appeal to students, in itself underpinning their creative and cognitive abilities [13]. In addition, positive correlations were identified between the level of VR integration and the frequency of VR use, while lower levels of VR integration were associated with more traditional teaching approaches [14].

National curriculum frameworks in Ireland’s second-level education sector have begun to recognize the unique impacts that digital technology can contribute to student learning. Following on from changes to the JC curriculum, in 2025, a new SC (Leaving Certificate) biology syllabus will be deployed which directly promotes student engagement with digital technologies to help ‘visualise, predict, explain, and model the organization, structures, processes, and interactions of living things’ [15]. However, the syllabus does not provide guidance on effective alignment of available digital technologies with targeted learning outcomes. Student understanding of cellular diversity across kingdoms, key subcellular macromolecules and integrated processes is strongly emphasized in this syllabus. However, in previous surveys of undergraduate microbiology students, 91% of respondents identified molecular biology concepts and spatial visualizations as the most challenging components of the curriculum [8,10]. Intervention with bespoke VR simulations, allowing student-paced, immersive interactions with cellular and sub-cellular components, was found to have significant positive impacts on student understanding [8,10]. Specifically, the interactive and visual elements of the simulations were found to be beneficial to learning, whereby users were tasked with the assembly of expression plasmids for recombinant protein production, selecting from core and decoy pieces, followed by assessment through Multiple Choice Questions and a visual simulation of the recombinant protein production process. In our view, VR immersion for cellular and molecular biology learning may represent an ideally aligned digital technology for such a purpose. Furthermore, VR application in second-level educational settings could be more impactful, by providing strong spatial modelling during initial student conceptualization of related curriculum. However, while the pedagogical and technological parameters may be well defined, an evaluation of logistical and practical challenges to effective deployment in second-level teaching settings would be required.

To address these gaps in institutional and sectoral knowledge, here, secondary school teachers and higher education scholars have reviewed the literature and brought their own expertise and reflections to explore the educational landscape and roadmap for the effective integration of immersive VR in the learning experience of STEM students. To provide a digital context and VR reference point through which to engage, teachers in mainstream, DEIS (Delivering Equality of Opportunity In Schools) and Gaelcholáiste (teaching through the medium of Irish) schools were provided with bespoke immersive VR simulations of animal, plant and bacterial cells, and Oculus Quest Head Mounted Displays (HMDs) through which to visualize them. This purposefully broad and inclusive approach gave us a snapshot reflecting the diversity of classrooms in the Irish education system. Together, we considered the practical challenges that need to be overcome before immersive VR can be readily integrated within the student learning experience. Equally, we consider the many opportunities for simulation development and classroom experiences.

## Design of immersive VR simulations and curriculum considerations

We have previously shown that abstract concepts in molecular and cellular sciences present a challenge to students insofar as they have little or no experience with 3D spatial representations or laboratory-based structural interactions through which to develop accurate and appropriate mental models. To begin this process, we developed a series of immersive virtual simulations based on the bacterial, human and plant cells (building on previous experience in molecular science simulation development [8,10]) and engaged with second-level teachers in Munster, Ireland, over a period of 2 years. The simulations present a visual and interactive representation of the cell for students to explore, either in class or in their own personal time, with key information identifying and explaining the function of core elements. While there is currently no assessment function within the simulations, and they are designed as a form of ‘learning by doing’, future iterations can integrate assessment ‘of’ or ‘for’ learning. Similarly, with the 3D-printed models, layering of assessment on the engagement would be quite easily achieved. The teachers were partners in this endeavour, providing guidance and feedback as the simulations were designed and developed. Their class sizes averaged between 25 and 30 students, ranging in age from 14 to 16 as part of the JC through the TY in second-level education. From the perspective of pedagogical implementation, a key element of this work was also to explore how the VR experience could be interwoven into the unique and challenging classroom learning spaces, particularly where resource and time management are such that HMD user time will be limited for each student within the classroom/lesson.

**Fig. 1.**
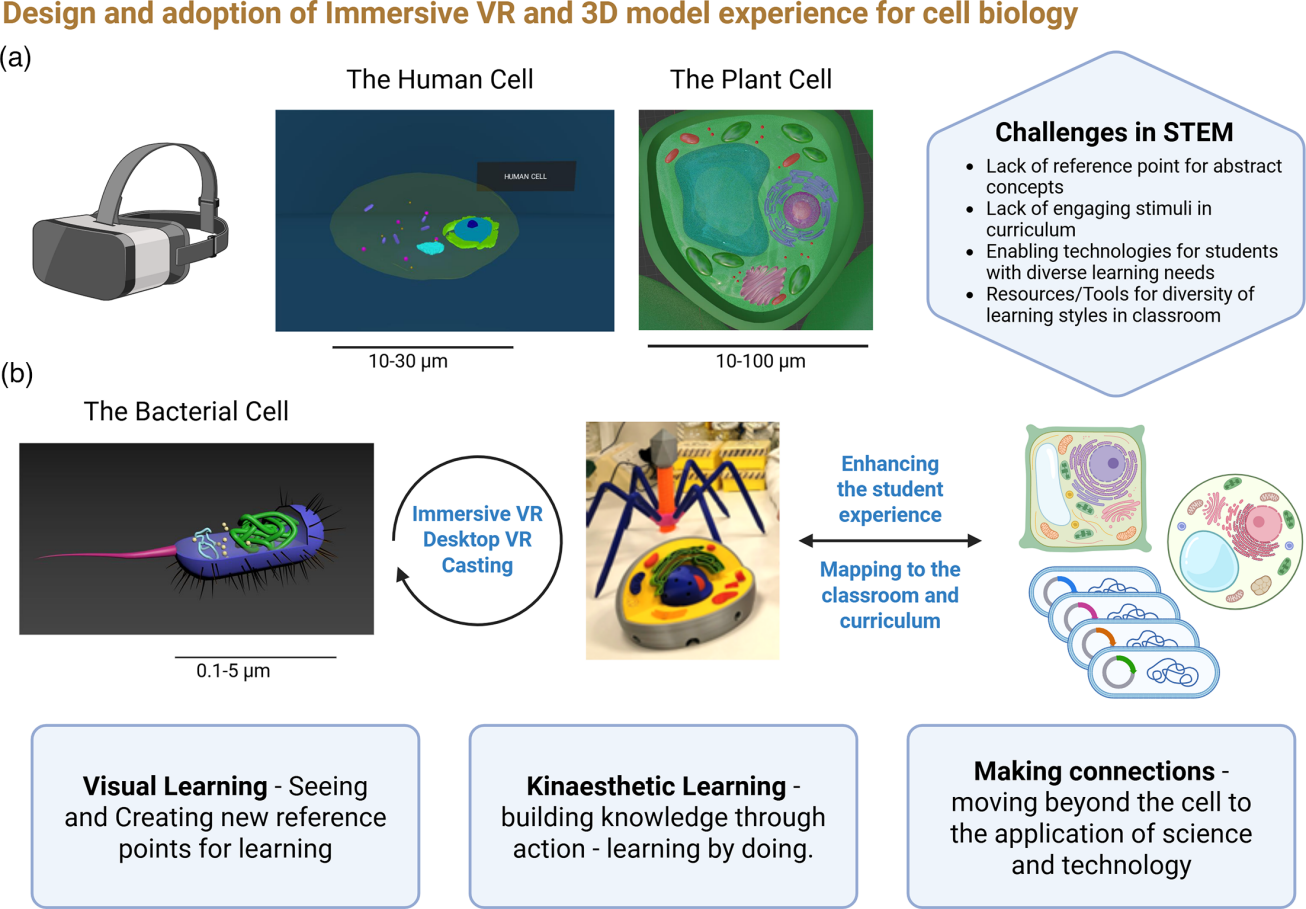
Overview of the classroom experience in an immersive VR environment, integrating new simulations with existing interactive and group-based classroom experiences. It is notable that immersive VR does not replace the tutor but rather acts to enhance the learning experience, providing new entry points for students through which to engage with STEM. (a) Screenshots of the human (animal) and plant cellular images used in the simulations show the simplicity of the visualizations, whereby interactivity and focus on key curriculum-related content were key design considerations. Challenges faced by students in accessing life science concepts and theory are presented. (b) Using the prokaryotic (bacterial) cell simulation as a case study, users engage in an iterative manner with the VR simulations and 3D-printed models, combining elements of visual and kinaesthetic learning. Mapping these experiences to ‘follow-on’ or ‘linked’ aspects of the curriculum serves to foster a deeper learning and connected understanding. The simulations presented here are accessed through separate links, and scale bars are included here to give a sense of average size. Image created using BioRender.com.

The VR simulations were web-hosted through Azure, which enabled sharing directly with teacher partners and accessed through the HMD internet browser. They were purposely designed to be scalable in the context of users and levels of immersion, interactive, multi-modal and dynamic. A multi-lingual platform was also developed to underpin these simulations, providing access in the desired language of learning for users. This is central to the use of VR in Gaelcholáiste, which teaches through the medium of the Irish language. In tandem with the immersive VR simulations, 3D-printed models were created in partnership with the library at University College Cork (Fig. 1), providing a tactile, build-and-learn tool which can be used in parallel with the immersive VR.

## Opportunities and challenges in adopting immersive VR technologies

There are some immediately apparent benefits of immersive VR, and these include interactive, performative elements of the experience through novel visual and spatial engagement with complex cellular entities. These new immersive digital platforms provide a kinaesthetic basis for interactions and skills for all users and may also support students who are less technologically literate with desktop environments while exploring complex scientific systems (Table 1). Nonetheless, these digital technology benefits are only significant if they deliver a tangible enhancement of student learning, clearly aligned with specific curriculum learning aims and objectives [16]. They must also represent deployable digital technologies for teachers, with robust guidelines for implementation to complement extant teaching and learning strategies. The authors acknowledge that financial constraints and both teacher and user competence will present significant challenges to implementation in certain contexts and settings. However, we are confident that this approach will become more mainstream and receive greater support as society continues to progress down the ‘digital yellow brick road’. Nuances around the language used in the simulations and the importance of making connections between what is learnt in the classroom and what is presented in the immersive simulations are also key considerations. While these are all factors that need to be accounted for before immersive VR can be successfully integrated within the second-level learning experience, some solutions are already available. The cost of HMDs has dropped considerably since their original introduction, such that it is now feasible for second-level schools to purchase multiple HMDs. The immersive VR simulations described here can form the basis of a suite of simulations, whereby the HMDs can be used widely within the curriculum, once a standardized practice of use has been established. Peer-to-peer learning through HMD is becoming possible, and this could create a new dynamic for students in second level, enabling remote student-paced learning that mirrors gamified interactions. Most recently, the use of generative artificial intelligence (AI) has brought excitement and tension in equal measure, as educators and learners strive towards a balance between technology-assisted and technology-delivered performances of understanding.

**Table 1.** Teachers' perspectives on opportunities, challenges and solutions facing adoption and implementation of VR in second-level education

Benefits for learning	Challenges to integration	Solutions to bottlenecks
Students get an interactive and immersive experience of the cell.	Ensuring it links to the JC/LC specification in more than one area.	Work in partnership to design the simulations, ensuring the best fit with the existing curriculum.
Incorporating technologies relevant to young adolescents makes the experience engaging and relatable. Students are using ‘cool’ technology that is different from usual methods, effectively a great stimulus to engage. VR complements the trend of Information and Communication Technology (ICT) push through the lens of engagement by increasing kids’ levels of engagement in their learning.	Limited access to HMDs within the classroom, need to have a programme for other students to do while HMDs are in use. Can be a long waiting time for students where numbers of headsets are low.	Activities for students not using the headset to scope outside the direct content of the simulation. Create a bank of resources/lessons around the cell possibly linking in with the microscope. Cast one student’s view onto the white board so all students get to see what the student is looking at. Having more VR headsets. With future investment/funding to increase the number of headsets, it could be used as a great opportunity for peer teaching/learning.
Opportunities to extend students' knowledge and grasp concepts of microbiology and the cell.	Language used at all levels. Definitions/explanations given are at lower to higher order phrasing.	Bring students to universities on a trip, so that they can connect learning to real-life applications of science. Create a slider in the simulations which offers more in-depth definitions and explanations. Students can then select the option that works for their knowledge/language level.
Use with first-year students to introduce the cell, VR would provide a visual reference point for students for what is essentially an abstract concept at this stage of their scientific learning journey. Students can spend time on it themselves and explore at their own pace.	Teacher competence using the VR headset will be a barrier, and student competence should not be assumed. Students who are less technologically literate could struggle.	Time allocation to provide training to teachers and students on how to use the technology and how to navigate the simulations.
The VR headset empowers our English as an Additional Language (EAL) students to engage with the curriculum, fostering a sense of belonging in Irish classrooms. Regardless of their English proficiency – whether level 1 or level 2 – students will learn and benefit from the visual biological images.	Level 1 EAL students may find it challenging to navigate the VR experience due to their limited English proficiency.	Simulations are made available on a multi-lingual platform. In the future, level 1 students with limited English might even translate the content into their native languages.
There is a strong push in the field of education with ICT. VR aptly fits for this push. Students are generally well-versed in the functioning of technology, not starting from scratch.	A major challenge is the learners and teachers’ ability to make connections between prior knowledge and new knowledge and when to apply or use VR in learning scenarios. Bridging the gap between past schemas (ways of thinking) and new/current schemas.	Ensure that the students can situate the VR experience in their own learning context, and create links to the classroom mental models accrued.
The use of the VR headset can allow our Junior Certificate School Programme (JCSP) students to engage in our lessons in a fun, exciting manner.	Having limited content on the VR.	Create content which incorporates more Priority Learning Units for our JCSP students.
Differentiation and assessment are great tools to differentiate for learners of mixed ability or weaker learners and to assess the needs of these students both in formative and summative assessment.	Very broad range of skill/expertise amongst the student body. How to ensure that one does not assess technical ability, where scientific knowledge and understanding are the goal.	Simplifying the VR simulation such that the controls are easily manipulated and technical ability is less of a factor in demonstrating learning.
VR facilitates and consolidates learning in relation to the learning styles of students (visual, tactile, etc.) and types of intelligence (theory).	The ability to know when an opportunity to connect new learning with prior knowledge using VR.	Use of storytelling and engaging stimuli such as images of videos and other interactive ways of building students’ interest in topics. This could also be solved through modelling (Bandura’s theory of observational learning) whilst using VR.

Some of the benefits for students are related to those with sensory needs, particularly for those who may require movement breaks. Several factors were identified in this aspect of teaching that are worth noting: **Controlled Environment** – VR allows for the creation of controlled environments where students can engage in activities that suit their needs without the unpredictability of the outside world. For students with autism, this can reduce anxiety and help them feel more secure while exploring different scenarios [17,18]; **Movement Breaks** – VR can be designed to include physical activities that encourage movement. For example, students can engage in games or simulations that require them to move around, stretch or perform physical tasks. This can provide a much-needed break from traditional classroom settings, helping to reset their focus and energy levels. By simply walking around the inside of a cell, they are both learning and achieving this break, all while being shut off from outside distractions; **Sensory Regulation** – many students with autism experience sensory overload. VR can help in sensory regulation by allowing students to immerse themselves in calming or stimulating environments tailored to their sensory preferences. For instance, they can explore tranquil scenes or participate in interactive activities that are visually and audibly soothing; **Motivation and Engagement** – the immersive nature of VR can captivate students' attention and motivate them to participate in learning activities. This can be particularly beneficial for students with autism who may struggle with traditional learning methods. The gamification of learning through VR can make educational content more appealing and enjoyable; **Physical Therapy and Movement** – for students who may have physical challenges, VR can be used in physical therapy to encourage movement in a fun way. Programmes can be designed to promote movement through interactive games, helping students improve their motor skills while enjoying the experience; **Feedback and Progress Tracking** – many VR systems can provide immediate feedback on a student’s performance, allowing for real-time adjustments and encouragement. This can help students understand their progress and areas for improvement, fostering a growth mindset.

While many of these challenges relating to the implementation of VR are encountered irrespective of the teaching environment, it should be noted that the integration of immersive VR experiences in second-level education also poses challenges that are not typically encountered in higher education. Principal amongst these is the role of the teacher in a classroom of students and limited access to HMDs through which to engage with the VR simulation. Therefore, the design of the classroom experience through which the VR will be used in parallel needs careful consideration (Table 1). For the immersive VR to have the most value, teacher training, student training and the ancillary tasks for students waiting their turn must all be factored in. In that sense, 3D models and other interactive experiences can be developed, such that the immersive VR experience becomes a ‘station’ within a broader classroom task-oriented experience.

**Table 2.** Organizing the integration of VR in the classroom experience

Design element	Details
Designated VR space	The classroom should have a specific area dedicated to VR activities. This space should be large enough to allow students to move around safely without obstacles.
Flexible seating arrangements	The classroom should feature flexible seating arrangements that can be easily reconfigured. This allows for group work, individual VR sessions or collaborative projects.
Technology integration	The classroom should be equipped with the necessary technology to support VR use. This includes high-speed internet access, charging stations for devices and a reliable power supply.
Curriculum alignment	The integration of VR should align with the curriculum. Teachers can select VR experiences that complement the topics being taught, whether it be virtual field trips or simulations of scientific processes. This ensures that VR is used as a tool for enhancing learning rather than as a stand-alone activity.
Training and support for educators	Teachers should receive training on how to effectively use VR in the classroom. This includes understanding how to set up the technology, troubleshoot issues and guide students in using VR experiences. Ongoing support should be available to help educators integrate VR into their lesson plans.
Scheduled VR sessions	VR should be integrated into the daily or weekly schedule. This could be structured as specific time slots for VR activities, allowing students to look forward to these sessions. Scheduling can include time for preparation, VR exploration and reflection or discussion afterward.
Reflection and feedback	After VR experiences, it is essential to have a structured reflection time. Students can discuss what they learnt, share their thoughts on the experience and provide feedback on the VR activities. This helps reinforce learning and allows teachers to adjust future VR content based on student input.
Accessibility considerations	The VR setup should be inclusive and accessible to all students. This includes considering the needs of students with different abilities and ensuring that the VR content is suitable for diverse learning styles. Adaptations may be necessary to accommodate sensory sensitivities or physical limitations.
Parental involvement and communication	Keeping parents informed about the use of VR in the classroom can foster support and understanding. Schools can host informational sessions or send newsletters explaining how VR enhances learning and what parents can do to support their children’s engagement with this technology.

## Perspectives on the integration of immersive VR within the classroom experience

Integrating VR as an educational tool in a classroom requires careful planning and restructuring to create an effective learning environment. Some key considerations for the design of space and time are outlined in Table 2. Alignment with the curriculum is highlighted as a design element of particular importance, notwithstanding the work required to create bespoke simulations that align with the needs of an evolving curriculum and educational landscape. This could also deliver meaningful interventions where a wider variety of content in the VR could offer a welcome distraction for students who often need movement breaks when feeling overwhelmed by the classroom curriculum.

It is clear from the breadth of elements discussed here that structuring a classroom to integrate VR as an educational tool involves creating a dedicated space, ensuring technological readiness, aligning with the curriculum, providing training for educators and fostering an inclusive environment. By thoughtfully integrating VR into the classroom, educators can enhance student engagement, promote interactive learning and provide unique educational experiences that cater to diverse learning needs. Microbiology presents an excellent example of where visualization of abstract and challenging molecular concepts can be presented to students in an accessible, interactive and visual way. Provision of the bacterial cell (including plasmid DNA) as a stand-alone simulation through the same point of access as the plant or animal cell offers a mechanism through which students can engage with microbiology material in a wider context. It can provide a means for comparison between the cell types, the immersive and 3D spatial aspect of this being of particular importance here. Indeed, promoting the study of microbiology within the science curriculum is an important endeavour, and one that can foster interest within the student population as they progress towards third-level education and/or future careers.

## Alignment with national and European policy on digital education and digital skills

The development and integration of VR simulations within second-level education should also be considered in the context of the national and European policies that call for and support the implementation of digital technologies in schools and the development of associated digital skills to empower learners.

At the national level, Ireland’s National Skills Strategy 2025 recognizes that technology is one of the key drivers of change in society and highlights the need for graduates with ICT for the future workplace [19]. In the Irish Department of Education’s Digital Strategy for Schools to 2027, the strategy’s vision highlights the need to ‘Empower schools to harness the opportunities of digital transformation to build digital competence’ [20]. The provision of VR headsets and the training on the use of VR, along with the associated secondary digital learnings, both tap into the two priority areas of the strategy of ‘Developing a high performing digital ecosystem’ and ‘Enhancing digital competences for the digital transformation’.

The targeting of DEIS schools in this study also aligns with the strategy's aims of facilitating equality of opportunity in education for all students. Digital technologies such as VR are highlighted in the document for their potential to promote and facilitate inclusion and accessibility, especially the educationally disadvantaged and learners with additional needs.

At the European level, the activity within this study aligns with the EU’s Digital Education Action Plan (2021–2027) Priority 1: Fostering the development of a high-performing digital education ecosystem, and the actions around inclusive primary and secondary education, and digital transformation plans for education and training institutions [21].

## Future directions and research questions

The advent of immersive VR in education and the development of next-generation display systems is promising, and yet the implementation of these technologies has not kept pace with the level of development in other areas such as AI. There is a range of factors that have contributed to this, including adequate resourcing, targeted staff training, constraints relating to curriculum-specific material and the importance of aligned assessments to name a few. In part, understanding the user-learning environment dynamic and where immersive VR fits within the personalized learning journey remains to be ascertained.

The next phase of this work will be to develop a research study to better understand the best mechanisms through which to deliver these simulation-based learning experiences in second-level education. With teachers as partners in this future research endeavour, the student voice will be an important dynamic in understanding what works, what doesn’t and what can be improved on such that the learning experience for these young scientists is optimal. It must also be remembered that the adoption of immersive VR in second-level education happens within a framework of curriculum and examination expectations on both teacher and student, and the integration of new learning modalities must be carefully managed not to upset the balance of the classroom and the diversity of student needs therein.

Key questions for consideration going forward will be:

1. Where best can immersive VR simulations enhance the student learning experience?
2. How best can immersive VR simulations enhance student engagement in STEM?
3. What are the key characteristics of optimized classroom experiences which integrate immersive VR and other performative learning activities?
4. How is the alignment of assessment and second-level curriculum learning outcomes supported in such integrated learning sessions?

This list of research questions is not exclusive but rather points to some of the key considerations that need to be addressed before immersive VR simulations could become a feature of the STEM learning journey in second-level education. Furthermore, in answering these questions, Hamilton *et al*. point to the need for a rigorous methodological approach through the identification of appropriate assessment measures, intervention characteristics and learning outcomes before any conclusions can be drawn on the application of immersive VR as a pedagogical method [3].

## Abbreviations

AI: artificial intelligence
DEIS: Delivering Equality of Opportunity In Schools
EAL: English as an Additional Language
HMDs: Head Mounted Displays
ICT: Information and Communication Technology
JC: Junior Cycle
JCSP: Junior Certificate School Programme
SC: Senior Cycle
STEM: science, technology, engineering and mathematics
TY: Transition Year
VR: virtual reality
